# Malaria during Pregnancy: Epidemiology, Current Prevention Strategies, and Future Directions^1^

**DOI:** 10.3201/eid1011.040624_09

**Published:** 2004-11

**Authors:** Robert D. Newman, Magda Robalo, Isabella Quakyi

**Affiliations:** * Centers for Disease Control and Prevention, Atlanta, Georgia, USA;; † World Health Organization/AFRO, Harare, Zimbabwe;; ‡ School of Public Health, College of Health Sciences, University of Ghana, Legon, Ghana

**Keywords:** malaria, Plasmodium falciparum, pregnancy, prevention, placenta, low birth weight, anemia, sulfadoxine-pyrimethamin

Malaria during pregnancy is a major public health problem in sub-Saharan Africa and is associated with maternal anemia, low birth weight, and premature delivery. Efficacious prevention measures exist, namely, insecticide treated bednets and intermittent preventive treatment. Malaria remains a disease of poverty; the fact that poor women are also less likely to revive antenatal care worsens the opportunities for intervention for the most vulnerable women. Future research needs to focus on new drugs and drug combinations for use in pregnancy, the impact of combined use of insecticide-treated bednets and intermittent preventive treatment, and the further exploration of a pregnancy-specific malaria vaccine.

## Epidemiology of Malaria during Pregnancy

Malaria during pregnancy poses substantial risk to the mother, her fetus, and the neonate; the infection contributes to as much as 15% of maternal anemia, 14% of low birth weight infants, 30% of preventable low birth weight, 70% of intrauterine growth retardation, 36% of premature deliveries, and 8% of infant mortality. In areas of stable transmission where adult women have considerable acquired immunity, *Plasmodium falciparum* infection during pregnancy typically does not cause symptomatic malaria but may lead to maternal anemia and placental malaria, especially among women having their first and second children. This placental malaria contributes to low birth weight, the single greatest risk factor for neonatal death, and a major contributor to infant deaths. In areas of unstable transmission, women do not acquire substantial antimalarial immunity; infection with *P. falciparum* can cause severe clinical illness and has also been linked to poor birth outcomes, including stillbirth and premature delivery. HIV infection diminishes a pregnant woman’s ability to control *P. falciparum* parasitemia and may contribute to up to 25% of maternal malaria, as well as contributing independently to maternal anemia, low birth weight infants, and infant deaths. The role of placental malaria in the vertical transmission of HIV remains unclear.

## Prevention of Malaria during Pregnancy and Its Adverse Consequences

Data from clinical trials and program evaluations in stable transmission areas indicate that intermittent preventive treatment with two doses of sulfadoxine-pyrimethamine is safe, efficacious, and effective in preventing maternal anemia, placental parasitemia, and low birth weight. Data also show that women using insecticide-treated bednets during pregnancy are also less likely to suffer these same adverse outcomes of malaria during pregnancy. The World Health Organization currently recommends that all pregnant women in malaria-endemic areas sleep under an insecticide treated bednet and receive at least two doses of intermittent preventive treatment with an efficacious antimalarial in the second and third trimesters. Chemoprophylaxis with chloroquine is no longer recommended. HIV-positive women need more than two doses of intermittent preventive treatment to achieve the same protective effect. Currently, the recommended drug for intermittent preventive treatment is sulfadoxine-pyrimethamine, because it is safe, easily delivered, and remains efficacious for women of reproductive age in most of sub-Saharan Africa.

## Future Directions in the Prevention of Malaria During Pregnancy

Because resistance of *P. falciparum* to current drugs and resistance of malaria vectors to available insecticides are both spreading, alternative strategies for the prevention of malaria during pregnancy need to be explored. The safety and efficacy of new antimalarial agents and antimalarial combinations for use in pregnancy require urgent investigation. The efficacy of current interventions in combination (i.e., insecticide-treated bednets together with intermittent preventive treatment) also merits further study. In addition, novel control mechanisms, such as a pregnancy-specific vaccine, should also be investigated. Potentially, a vaccine could be developed that could protect against placental sequestration and the associated adverse effects. Until such a vaccine is developed, insecticide-treated bednets and intermittent preventive therapy will remain the mainstay of malaria control efforts during pregnancy.

